# Oncological Outcomes of Concurrent Chemoradiotherapy and Surgical Treatment for Patients With T3 Hypopharyngeal Cancer: A Single-Center Retrospective Analysis

**DOI:** 10.7759/cureus.62553

**Published:** 2024-06-17

**Authors:** Mitsuko Yui, Yoshihisa Matsuno, Tatsuya Furukawa, Masanori Teshima, Hirotaka Shinomiya, Naomi Kiyota, Tadashi Nomura, Daisuke Miyawaki, Ryohei Sasaki, Ken-ichi Nibu

**Affiliations:** 1 Otolaryngology - Head and Neck Surgery, Kobe University Graduate School of Medicine, Kobe, JPN; 2 Medical Oncology and Hematology, Cancer Center, Kobe University Graduate School of Medicine, Kobe, JPN; 3 Plastic Surgery, Kobe University Graduate School of Medicine, Kobe, JPN; 4 Radiation Oncology, Kobe University Graduate School of Medicine, Kobe, JPN; 5 Radiation Oncology, Kobe University Hospital, Hyogo, JPN

**Keywords:** adverse event, postoperative complication, pharyngolaryngectomy, salvage surgery, chemoradiotherapy

## Abstract

Background

Since the larynx and pharynx are vital for respiration, swallowing, and speech, chemoradiotherapy (CRT) has been widely applied for T3 hypopharyngeal cancer (HPC) as an organ-preserving treatment. However, CRT can lead to severe late adverse events such as dysphagia and aspiration pneumonia, especially in patients who have difficulty swallowing and/or aspiration at the time of initial diagnosis.

Patients and methods

Between 2012 and 2020, 86 patients with T3 HPC treated with curative intent at Kobe University Hospital were included in this study. The average age was 69 years old, ranging from 43 to 89. Diseases were classified as Stage III in 29 patients, Stage IVA in 52 patients, and Stage IVB in five patients. Thirty-five (41%) patients were treated by CRT, and 51 (59%) patients were treated by surgery. Patients were followed up for at least two years, and the follow-up period ranged from four to 128 months (median: 45 months).

Results

Three-year progression-free survival (PFS) rates of patients treated by CRT and patients treated by surgery were 56.2% and 60.3%, respectively. Three-year disease-specific survival (DSS) rates of patients treated by CRT and surgically treated patients were 79.0% vs. 70.8%, respectively. Three-year overall survival (OS) rates of patients treated by CRT and surgically treated patients were 64.5% and 69.0%, respectively. Of note, a significant difference was observed between three-year DSS and three-year PFS (79.0% vs. 56.2%, p = 0.0014) in the patients treated by CRT but not in the patients treated by surgery.

Conclusions

No significant differences were observed between the PFS, DSS, and OS rates of patients treated by CRT and those of surgically treated patients. Locoregional recurrences after CRT were significantly successfully salvaged by surgical intervention. These results suggest that CRT can be applied as an alternative to surgery without reducing survival, especially for patients without severe clinical symptoms. Meticulous follow-up is mandatory for early detection of recurrence to salvage by surgery and for the management of late adverse events.

## Introduction

Hypopharyngeal cancer (HPC) has the worst prognosis among head and neck cancers in Japan [1,2], with a five-year disease-specific survival (DSS) rate of 1,321 (59.3%) out of 2,229 patients [3]. Histologically, the majority of HPC is squamous cell carcinoma [1], and alcohol consumption has been reported to be related to an increased risk of HPC [4]. Alcohol is initially oxidized to acetaldehyde by alcohol dehydrogenase 1B, then to a well-known carcinogen, acetic acid, by aldehyde dehydrogenase (ALDH2). The gene-encoding ALDH2 exhibits polymorphism and ethnic variation. While ALDH2*1 has normal activity, ALDH2*2 has markedly reduced enzymatic activity, resulting in an accumulation of toxic ALDH. Since ALDH2*2 is the dominant negative, drinkers with heterozygosity of ALDH2*1 and ALDH2*2 are at significantly high risk of alcohol-related cancers within the upper aerodigestive tract, such as oropharyngeal, hypopharyngeal, and esophageal cancers. While most Caucasians have homozygous ALDH2*1, about half of the Japanese have heterozygous ALDH2*1 and ALDH2*2 [4,5]. Reflecting these backgrounds, HPC is the second most common head and neck cancer in Japan, accounting for about 3,047 (23%) out of 13,390 head and neck cancers, with an estimated annual incidence of 4:100,000 in 2020 [1]. In addition, despite the widespread periodical health checkup system, including gastrointestinal endoscopy, and enthusiastic awareness-raising activities by academic societies and various organizations, 1,144 (62.4%) of 3,047 HPC are still diagnosed at advanced stages (stage III/IV) [1].

Since the larynx and pharynx are vital for respiration, swallowing, and speech, it is required both to control disease and maintain quality of life in the treatment of advanced HPC. To address this issue, chemoradiotherapy (CRT) has been recommended and widely applied for T3 HPC as a larynx-sparing treatment and has provided favorable oncological and functional outcomes [6]. However, CRT can lead to several severe late toxicities, such as dysphagia and aspiration pneumonia, especially in patients who have difficulty swallowing and/or aspirating at the time of initial diagnosis [2,7]. In the event of local and/or regional recurrence, salvage surgery is often associated with severe postoperative complications due to the fibrosis of the tissues induced by irradiation [8,9].

To explore the optimal treatment strategy for individual patients with T3 HPC, in this study, we retrospectively reviewed the oncological outcomes of the patients with T3 HPC, either treated by upfront surgery or CRT with curative intent, and examined the appropriateness of our choice of treatment for the patients with T3 HPC.

## Materials and methods

Between 2012 and 2020, 344 patients with HPC were treated with curative intent at Kobe University Hospital. The extent of the tumors was closely examined by enhanced CT and fluorodeoxyglucose-PET (FDG-PET). Primary sizes were measured as the maximum major axis and minor axis based on the axial section of enhanced CT. Also, all patients underwent upper gastrointestinal endoscopy to evaluate the extent of HPC and to investigate simultaneous esophageal and/or gastric cancer. Consequently, 86 patients diagnosed as having T3 HPC were included in this study according to the UICC TNM Classification of Malignant Tumors, 8th Edition [10]. Patients with distant metastasis at the time of initial diagnosis and patients treated with chemotherapy alone or radiation with palliative intent were not included in this cohort. There were 79 males and seven females. The average age was 69 years old, ranging from 43 to 89. Diseases were classified as Stage III in 29 patients, Stage IVA in 52 patients, and Stage IVB in five patients. Nodal statuses were classified as N0 in 19 patients, N1 in 10 patients, N2a in five patients, N2b in 29 patients, N2c in 18 patients, N3a in no patients, and N3b in five patients. The primary subsite was the pyriform sinus in 63 patients, the postcricoid region in 13 patients, and the posterior wall in 10 patients. Patients were followed up for at least two years. The follow-up period ranged from four to 128 months (median: 45 months).

Principally, we recommended cisplatin (CDDP)-based CRT to patients with T3 HPC. In general, considering the average renal function of Japanese patients [11], 80 mg/m^2^ of CDDP was administered every three weeks, and 70 Gy of radiotherapy (RT) was administered in total (five times per week, 2 Gy per fraction) at clinical practice. The dose of CDDP was adjusted accordingly, depending on the renal function of each patient. For the patients who were not feasible with CDDP, carboplatin (CBDCA) or cetuximab (Cmab) was used as a concomitant agent with radiation. Neck dissection prior to CRT was recommended for patients who had neck metastasis firmly involving the internal jugular vein and/or sternocleidomastoid muscle [12], based on the approval of the head and neck cancer board.

For patients who were not feasible for CRT due to renal, liver, cardiac, pulmonary, and/or cognitive functions, we recommended surgical treatment. Surgical treatment was also recommended for patients with large volumes of primary tumor and/or with severe symptoms, such as difficulty swallowing, aspiration, and/or dyspnea requiring a tracheotomy. If the patients with these features refused surgical treatment, induction chemotherapy was recommended [13-15]. Postoperative CDDP-based CRT, or RT, was administered to patients at high risk of recurrence according to the Japanese Clinical Practice Guidelines for Head and Neck Cancer [6] and the NCCN Guidelines for Head and Neck Cancers [16].

This study was approved by the Ethical Committee of Kobe University Hospital (#1586), and written informed consent was obtained from all the patients. Clinical information was obtained from medical records. The progression-free survival (PFS) rate, disease-free survival (DFS) rate, and overall survival (OS) rate were illustrated using the Kaplan-Meier method. A log-rank test was used to analyze differences in survival. Characteristic differences between the patients treated by CRT and those treated by surgery were assessed with the chi-square test or Fisher’s exact test. All statistical analyses were performed using R version 4 (The R Foundation).

## Results

Treatment modalities of the 86 patients with T3 HPC

**Figure 1:**
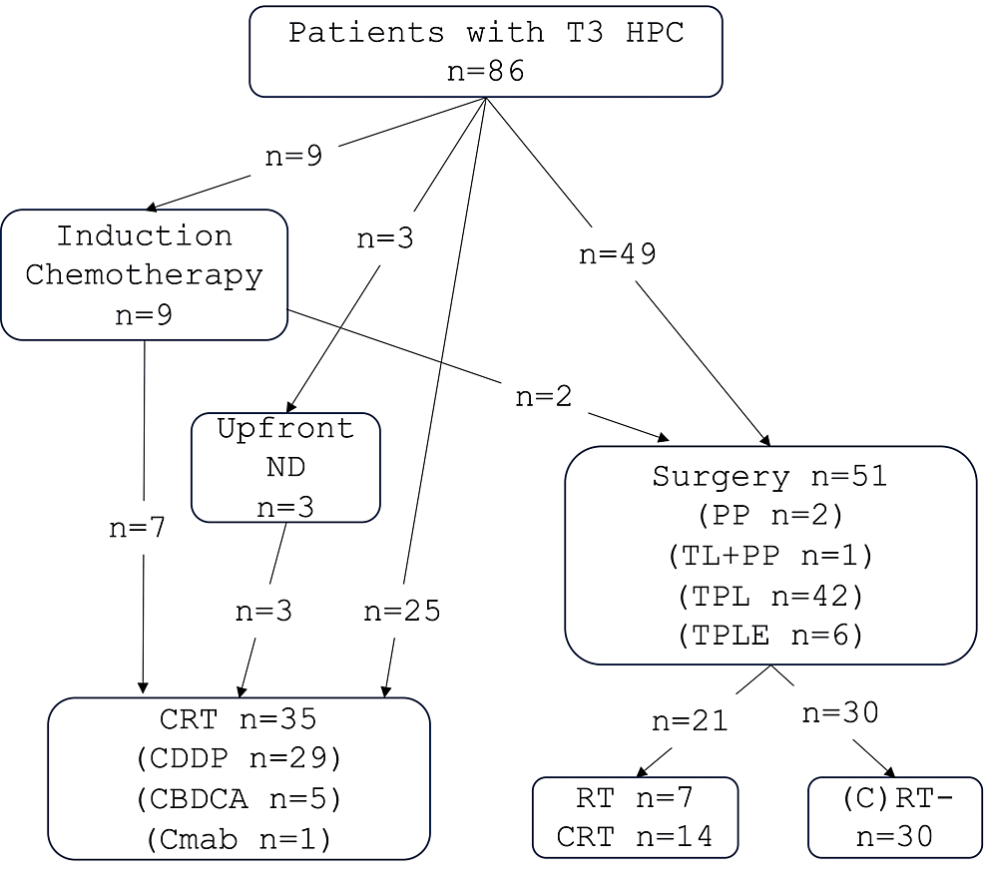
Treatments of the 86 patients with T3 HPC CBDCA, carboplatin; CDDP, cisplatin; Cmab, cetuximab; CRT, chemoradiotherapy; HPC, hypopharyngeal cancer; ND, neck dissection; PP, partial pharyngectomy; RT, radiotherapy; TL, total laryngectomy + partial pharyngectomy; TPL, total pharyngolaryngectomy, TPLE: total pharyngolaryngoesophagectomy

Among the 86 patients, induction chemotherapy was performed in nine patients: TPF (docetaxel, CDDP, and fluorouracil (5FU)) in eight patients, and the FP (5FU and CDDP) regimen in one patient. Among these nine patients, seven patients with favorable responses subsequently underwent CRT, and two patients with stable disease had surgical treatment. Finally, 35 (41%) patients were treated by CRT, and 51 (59%) patients were treated by surgery (Figure 1).

Among the 35 patients treated by CRT, three underwent ipsilateral neck dissection prior to CRT. The chemotherapeutic agents used with RT were CDDP in 29 patients, CBDCA in five patients, and Cmab in one patient. In the surgically treated 51 patients, the larynx was preserved in two patients who had a partial pharyngectomy. Partial pharyngectomy with total laryngectomy, total pharyngolaryngecomy, and total pharyngolaryngoesophagectomy was performed in one, 42, and six patients, respectively. Postoperative CDDP-based CRT and RT were performed in 14 and seven patients, respectively. The other 30 patients had no postoperative adjuvant therapy (Figure 1).

Characteristics of the patients treated by surgery and CRT

The characteristics of the patients according to the initial treatment modality are summarized in Table 1. Surgically treated patients were significantly older than those treated by CRT (71.9 years old vs. 65.4 years old, p = 0.001). Fixation of the vocal cord (38 (74.5%) vs. 18 (51.4%), p = 0.048), and dysphagia (7 (13.7%) vs. 0 (0%), p = 0.0215) were significantly more common in surgically treated patients. In addition, one-fifth of the surgical patients underwent a tracheotomy before surgery, while no patient treated by CRT had a tracheotomy (p = 0.0019). While statistical analysis did not reach significance, the size of the primary was larger, and renal function was poorer in the surgically treated patients in comparison with patients treated by CRT. No significant difference was observed among the subsites between the surgically treated patients and patients treated by CRT.

**Table 1:** Characteristics of the patients treated by surgery and CRT CRT, chemoradiotherapy; eGFR, estimated glomerular filtration rate; PC, post cricoid region; PS, piriform sinus; PW, posterior wall

Parameter	Surgery (n = 51)	CRT (n = 35)	p	t-value	X^2^ value	OR
Average age (years old)	71.9	65.4	0.001	3.115	-	-
Vocal cord fixed/mobile	38/13	18/17	0.048	-	3.904	-
Primary size (major axis)	33 mm	24 mm	0.07	0.1433	-	-
Primary size (major axis)	16 mm	12 mm	0.43	0.169	-	-
PS/PC+PW	38/8+5	25/5+5	0.75	-	0.0994	-
N0-2a/N2b-3b	19/32	14/21	0.97	-	0.0009	-
eGFR <60/≥60	16/35	5/30	0.12	-	2.423	-
Preoperative tracheotomy +/-	11/40	0/35	0.0019	-	-	20.16
Dysphagia +/-	7/444	0/35	0.0215	-	-	11.97

Reasons for recommending surgical treatment based on medical records are summarized in Table 2. Severe dyspnea and dysphagia were the most common reasons to recommend surgery in 25 (49%) patients, followed by the history of RT for the treatment of previous head and neck cancer in six (12%) patients. Poor renal function, simultaneous advanced esophageal cancer, and advanced age were the main reasons in the respective three patients. Surgical treatment was also recommended for one patient with N3b [12] and one patient with metastasis in the retropharyngeal lymph node [17].

**Table 2:** Main reason for recommending surgery RPLN, retropharyngeal lymph node; RT, radiotherapy

Clinical factors	Number of patients
Severe dyspnea/dysphagia	25
History of RT in the neck	6
Poor renal function	3
Simultaneous esophageal cancer	3
Advanced age	3
N3b	1
Metastasis to RPLN	1
Others (mental disorder, collagen disease, and patient’s preference)	9

Oncological outcomes of the patients treated by CRT and surgery

**Figure 2:**
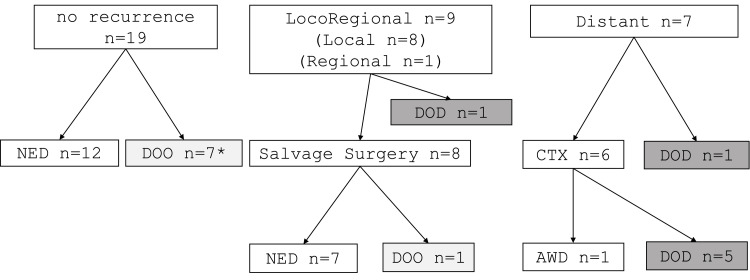
Oncological outcomes of the patients with T3 HP treated by CRT as initial treatment * Two of them died of aspiration pneumonia. CRT, chemoradiotherapy; CTX, chemotherapy; distant, distant metastasis; DOD, died of disease; DOO, died of other disease; HPC, hypopharyngeal cancer; local, local recurrence; NED, alive with no evidence of disease; regional, regional recurrence

The oncological outcomes of the patients treated by CRT are summarized in Figure 2. During the follow-up periods, locoregional recurrence was observed in nine patients. Among them, eight patients underwent salvage surgery. Distant metastasis developed in seven patients, and six out of these seven patients underwent chemotherapy. No locoregional or distant metastasis was observed in the other 19 patients. At the end of follow-up periods, among the 19 patients without recurrence, 12 were alive with no evidence of disease; two patients died of aspiration pneumonia, possibly due to the late toxicity of CRT; and five patients died of another disease. Among the nine patients who had local recurrence, seven patients have been alive without disease, one patient died of another disease, and one patient who did not have salvage surgery died of HPC. Among the patients with distant metastasis, one patient has been alive with the disease.

**Figure 3:**
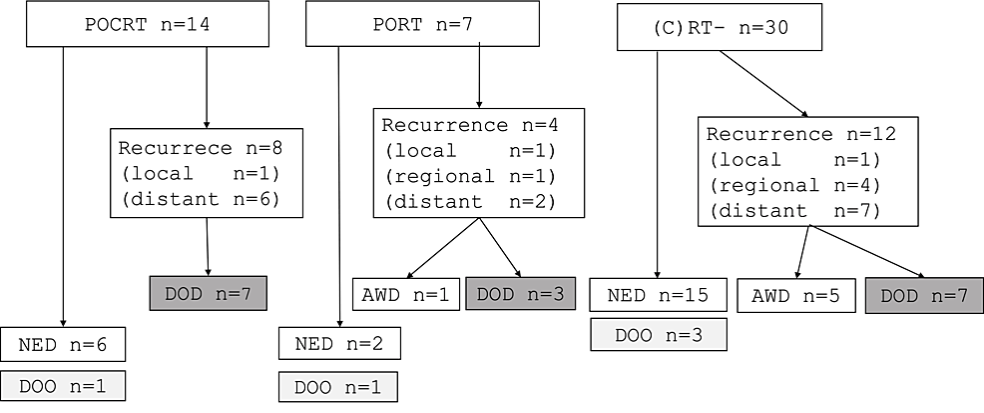
Oncological outcomes of the patients with T3 HPC treated by surgery as initial treatment AWD, alive with disease; (C)RT-, postoperative CRT or RT was not performed; distant, distant metastasis; DOD, died of disease; DOO, died of other disease or cause; HPC, hypopharyngeal cancer; local, local recurrence; NED, alive with no evidence of disease; POCRT, postoperative chemoradiotherapy; PORT, postoperative radiotherapy; regional, regional recurrence

The oncological outcomes of the patients treated by surgery are summarized in Figure 3. During the follow-up period, local and/or regional recurrence was observed in three (14%) out of 21 patients who had postoperative CRT or RT and five (16%) out of 30 patients without postoperative CRT or RT. Distant metastasis developed in 15 (29.4%) patients. At the end of the follow-up period, 23 patients were alive without disease, and five patients died of other causes. Among the 23 patients who had a locoregional recurrence or distant metastasis, six patients lived with the disease, and 17 patients died of HPC.

**Figure 4:**
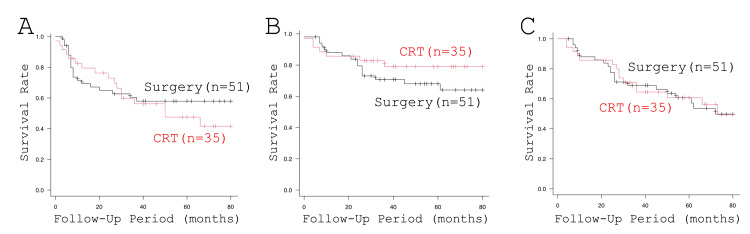
Survival of the patients according to the treatment modalities: (A) PFS, (B) DSS, and (C) OS CRT, chemoradiotherapy; DSS, disease-specific survival; OS, overall survival; PFS, progression-free survival; TPL, total pharyngolaryngectomy

**Figure 5:**
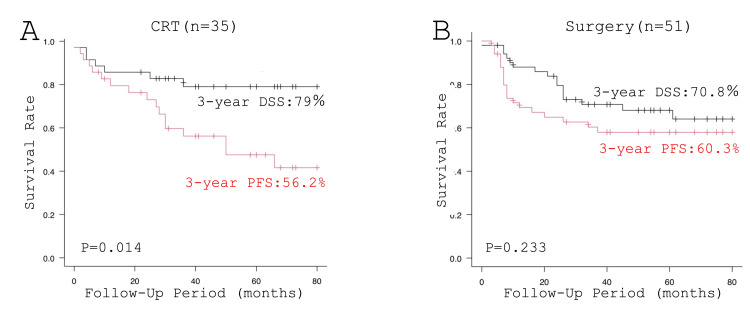
Comparisons of DSS and PFS of the patients treated by CRT and surgery: (A) CRT and (B) surgery CRT, chemoradiotherapy; DSS, disease-specific survival; PFS, progression-free survival

Finally, the survival curves of the patients treated by CRT and surgery as an initial treatment are illustrated in Figure 4. Three-year PFS rates of patients treated by CRT and patients treated by surgery were 20 (56.2%) and 31 (60.3%), respectively (p = 0.57). The three-year DSS rate of the patients treated by CRT tended to be better in comparison with the surgically treated patients (28 (79.0%) vs. 36 (70.8%), p = 0.27). Three-year OS rates of patients treated by CRT and patients treated by surgery were 23 (64.5%) and 35 (69.0%), respectively (0.57). Of note, a significant difference was observed between the three-year DSS rate and three-year PFS rate (28 (79.0%) vs. 20 (56.2%), p = 0.0014) in the patients treated by CRT but not in the patients treated by surgery (36 (70.8%) vs. 31 (60.3%), p = 0.233, Figure 5).

## Discussion

Reasons for the choice of treatment

Since the larynx and pharynx are vital for respiration, swallowing, and speech, CRT has been recommended and widely applied for T3 HPC as a larynx-sparing treatment [16]. Accordingly, in the United States, 240 (79%) out of 302 patients with T3 were treated with CRT as the first-line therapy [18]. However, in Japan, more than half of the patients with T3 HPC were treated by surgery [1,19], as seen in the present study.

By statistical analysis, surgically treated patients were significantly older than those treated by CRT. Owing to the advances in surgical techniques and perioperative management, as well as a well-developed public insurance system for the elderly, surgery for head and neck surgery requiring free flap reconstruction has been performed even in elderly patients in Japan [20]. On the contrary, CRT requires sufficient renal, liver, bone marrow, and cardiopulmonary functions to tolerate nephrotoxicity and a large volume of infusion. Also, appropriate cognitive function is mandatory to complete a long period of treatment. However, reflecting the most aged society in the world, now half of the patients with head and neck cancer are over 70 years old in Japan [1]. Thus, a considerable number of elderly patients with head and neck cancers are not feasible for CRT due to the decreased functions of these organs [21], as shown in the present study. The larger proportion of elderly patients with HPC might be the most possible reason why more patients with HPC have been surgically treated in Japan.

In the present study, a history of RT and simultaneous advanced esophageal cancer were also noted as reasons for recommending surgery. As mentioned in the introduction, about half of Japanese have heterozygous ALDH2*1 and ALDH2*2 and are at significantly high risk of alcohol-related head and neck and/or esophageal cancer [5]. According to the Report of the Head and Neck Cancer Registry of Japan, 354 (15.8%) out of 2,229 patients with newly diagnosed HPC had a history of metachronous head and neck or esophageal cancer at the initial diagnosis [3]. If previous head and neck or esophageal cancers were treated by RT of CRT, CRT could not be applied in these patients. The larger proportion of patients with heterozygous ALDH2 genes might be another possible reason why more patients with HPC have been surgically treated in Japan.

In the present study, patients treated by surgery were more likely to have fixation of vocal cords, dysphagia, and/or severe dyspnea requiring a tracheotomy compared with the patients treated by CRT. It has been reported that CRT makes preservation of laryngeal function difficult in patients with severe symptoms such as dyspnea and/or dysphagia at the time of initial treatment, even if the larynx is anatomically preserved [7,8,22,23]. In addition, tumor volume has been reported to highly predict local responses [13,24-26]. Accordingly, advanced locoregional diseases, especially those showing severe clinical symptoms, were the most common reason for recommending surgery in the present series. However, most patients who had induction chemotherapy showed a complete or partial response and were subsequently treated with CRT. Induction chemotherapy might be more proactively considered in patients with advanced locoregional diseases showing these severe clinical symptoms [13-15].

Oncological outcomes of the patients treated by surgery and CRT

In the United States, the OS of patients with T3 treated by surgery was significantly better compared with the patients treated by CRT [18]. On the other hand, according to a multicenter study in Japan, no significant difference was observed in the OS rates between patients treated by CRT and patients treated by surgery [19]. Accordingly, in the present study, the PFS of the patients treated by CRT and that of patients treated by surgery were similar. Moreover, the DSS of patients treated by CRT tended to be better compared with patients treated by surgery, partly due to the selection bias discussed above.

Of note, a significant difference was observed between DSS and PFS in the patients treated by CRT, suggesting a significant number of the patients were salvaged by surgery in comparison with the previous reports [9,27]. Since population density is high and the public transportation network is well developed in our territory, most of our patients periodically visit our hospital for follow-up and see their attending head and neck surgeons. In addition, in Japan, public insurance covers periodical CT, MRI, and FDG-PET, if indicated. We believe that these circumstances enabled meticulous follow-up and early detection of recurrence, leading to successful salvage surgery. On the other hand, treatment-related deaths (lung abscess and pneumonia) due to aspiration were observed in two (6%) of 35 patients treated by CRT. Long-term, careful follow-up, especially paying attention to dysphagia and aspiration pneumonia, is required in patients with HPC treated by CRT [7,8,14].

One of the limitations of the present study is the relatively small number of patients in the present series. Another limitation is the retrospective nature of this study, while it is ethically difficult to conduct a randomized study of CRT versus surgery requiring laryngectomy. To address these limitations, we are currently conducting matched pair analysis using the clinical data of over 1,000 patients with HPC registered in the Report of the Head and Neck Cancer Registry of Japan. This study will provide more definitive evidence to develop optimal treatment strategies for individual patients with T3 HPC.

## Conclusions

In this study, no significant differences were observed between PFS (56.2% vs. 60.3%), DSS (79.0% vs. 70.8%), and OS (64.5% vs. 69%) rates among the patients treated by CRT and those of surgically treated patients. Locoregional recurrences after CRT were significantly successfully salvaged by surgical intervention. These results suggest that CRT can be applied as an alternative to surgery without reducing survival, especially for patients without severe clinical symptoms. Meticulous follow-up is mandatory for early detection of recurrence to salvage by surgery and for the management of late adverse events.
